# Separating acoustic deviance from novelty during the first year of life: a review of event-related potential evidence

**DOI:** 10.3389/fpsyg.2013.00595

**Published:** 2013-09-05

**Authors:** Elena V. Kushnerenko, Bea R. H. Van den Bergh, István Winkler

**Affiliations:** ^1^School of Psychology, Institute for Research in Child Development, University of East LondonLondon, UK; ^2^Department of Psychology, Tilburg UniversityTilburg, Netherlands; ^3^Department of Psychology, Katholieke Universiteit LeuvenKU Leuven, Belgium; ^4^Department of Experimental Psychology, Institute of Cognitive Neuroscience and Psychology, Research Centre for Natural Sciences, Hungarian Academy of SciencesBudapest, Hungary; ^5^Institute of Psychology, University of SzegedSzeged, Hungary

**Keywords:** orienting, passive auditory attention, distraction, infants, event-related potential (ERP), novelty detection, oddball paradigm, mismatch negativity (MMN)

## Abstract

Orienting to salient events in the environment is a first step in the development of attention in young infants. Electrophysiological studies have indicated that in newborns and young infants, sounds with widely distributed spectral energy, such as noise and various environmental sounds, as well as sounds widely deviating from their context elicit an event-related potential (ERP) similar to the adult P3a response. We discuss how the maturation of event-related potentials parallels the process of the development of passive auditory attention during the first year of life. Behavioral studies have indicated that the neonatal orientation to high-energy stimuli gradually changes to attending to genuine novelty and other significant events by approximately 9 months of age. In accordance with these changes, in newborns, the ERP response to large acoustic deviance is dramatically larger than that to small and moderate deviations. This ERP difference, however, rapidly decreases within first months of life and the differentiation of the ERP response to genuine novelty from that to spectrally rich but repeatedly presented sounds commences during the same period. The relative decrease of the response amplitudes elicited by high-energy stimuli may reflect development of an inhibitory brain network suppressing the processing of uninformative stimuli. Based on data obtained from healthy full-term and pre-term infants as well as from infants at risk for various developmental problems, we suggest that the electrophysiological indices of the processing of acoustic and contextual deviance may be indicative of the functioning of auditory attention, a crucial prerequisite of learning and language development.

## Introduction

Auditory attention is a key prerequisite of acquiring many important skills, such as for example learning to speak and communicate with others. It is well known that very young infants have a natural attraction and early preference for speech sounds (Vouloumanos and Werker, 2007). However, there are also non-speech sounds that require one's attention in the auditory environment. Some of these sounds may signal an opportunity or some danger, while others may be irrelevant for the current behavioral goals. Therefore, it is important to determine which stimuli require further processing. Many potentially informative sounds result from the arrival (or activation) of a new object (sound source) in the environment. A typical common cue for detecting sounds originating from such new sources is that they often widely differ from the sounds previously encountered. Thus, one may expect the human auditory system to be sensitive to large acoustic deviations already at birth. However, acoustic deviance (salience) does not tell the full story. Therefore, an important early developmental goal is to separate acoustic deviance from relevant information.

Here we review studies testing the processing of sounds widely deviating from the preceding acoustic environment during the first year of life^1^. We focus on investigations recording event-related brain potentials (ERP) and magnetic fields (ERF) as this method is amongst the few consistently available throughout this period. Whereas the behavioral repertoire of infants undergoes rapid changes during the first year of life, brain responses can be measured for similar or even identical stimulus paradigms throughout the whole period. Further, few studies using alternative brain measures, such as near infrared spectroscopy (NIRS) and functional magnetic resonance imaging (fMRI) have yet been published

The layout of the review is as follows: (1) a short and limited description of the development of passive auditory attention during the first year of life together with the most typical ERP paradigm used to study this function; (2) the ERP components observed in the context of auditory deviance detection and attention switching subdivided into three periods: newborn, 2–6 month, and 6–12 month old infants; (3) discussion of the development of the ERP components during the three periods and issues for future research. Previous studies have demonstrated that these time periods correspond to milestones in neuroanatomical development: increase in synaptogenesis between birth and 3 months of age in auditory cortex (and by ~6 months in visual cortex) (Huttenlocher, 1984; Huttenlocher and Dabholkar, 1997), followed by a rise in cerebral metabolic rate (Chugani et al., 1987) and an increase of white matter in association cortices by ~8–12 months (Paus et al., 2001). We expect these important steps in brain maturation to be reflected in the morphology and functionality of the ERP responses elicited by widely deviant sounds.

## Development of passive auditory attention during the first year of life

The four components appearing in some current complete models of attention are arousal, orienting, selective and sustained attention (Posner and Petersen, 1990; Cowan, 1995; Ruff and Rothbart, 1996; Gomes et al., 2000). These components reflect the general state of an organism with respect to processing information and the abilities to orient toward, select, switch between, and maintain focus on some information source. Another distinction can be made on the basis of whether an attention-related change is triggered by some external event, such as involuntarily orienting toward a new sound source (termed stimulus-driven, bottom-up, or passive attention; James, 1890) or it is under the voluntary control of the organism (termed top-down or active attention). The current review focuses on the early development of the processes triggered by salient external stimuli, constituting the first steps of passive attention. These processes may lead to orienting. When orienting is triggered by some stimulus that is irrelevant to the ongoing behavior (e.g., a loud sound while one is reading a book), attention switches to this stimulus. This phenomenon is termed distraction. Finally, one may return to the original task if the stimulus did not signal something more important to deal with. This phenomenon is termed reorientation. The cycle of passive attention, attention switching, orientation, distraction, and reorientation provide the background for interpreting the results reviewed here.

The ontogenetically earliest manifestations of attention primarily include orienting toward stimuli of potential biological significance (for a review, see Gomes et al., 2000). This is the aspect of attention, which is also evoked by acoustic deviance. Orienting refers to the physiological and behavioral changes associated with the detection of a stimulus with some novel aspect. The orienting response (OR; Pavlov, 1927; Sokolov, 1963; Sokolov et al., 2002) is a combination of overt and covert responses associated with searching for and preferential processing of new information. Components of the OR are targeting responses (eye, hand, and body movements), autonomic reactions (cardiac and skin conductance response), desynchronization of the electroencephalogram (EEG), and augmentation of certain ERP components (Sokolov et al., 2002; Kushnerenko et al., 2007; Kushnerenko and Johnson, 2010).

In infants, orienting is most commonly assessed by spontaneous motor and psychophysiological responses: e.g., localized head turning (Clarkson and Berg, 1983; Morrongiello et al., 1994), or changes in the heart rate (Clarkson and Berg, 1983; Richards and Casey, 1991). Sometimes such parameters as behavioral inhibition, motor quieting, and eye widening are also used in assessing the responsiveness of newborns (Gomes et al., 2000). Orienting responses to various sounds (bell, rattle, voice) are often used in neonatal clinical assessments (Foreman et al., 1991; Riese, 1998; Van de Weijer-Bergsma et al., 2008). Neonatal reactivity to such sounds appears to be predictive of further development of temperament (Riese, 1998) and later attentional and behavioral functioning (for a review, see Van de Weijer-Bergsma et al., 2008).

Neonatal ERP responses to sounds are strongly influenced by stimulus parameters: newborns preferentially respond to broadband noise compared to tones (Turkewitz et al., 1972; Werner and Boike, 2001; Kushnerenko et al., 2007), to high compared to low frequency noise (Morrongiello and Clifton, 1984), to tones of longer rather than shorter duration (Clarkson et al., 1989). Acoustic features (such as intensity or spectral complexity) are apparently initially the most salient cues for passive attention and, as a consequence, orienting (Kushnerenko et al., 2007). It is important to note that passive auditory attention is strongly limited by the initially low frequency resolution of the infant auditory system until ca. the age of 6 months and even beyond by the immaturity of some higher-level auditory processes (Werner, 2007). An example of the latter is that differences in thresholds for pure-tone detection between infants and adults could be partly accounted for by differences in listening strategies: it has been suggested that young infants do not selectively focus on frequencies for discriminating sounds (Werner and Boike, 2001). One possibly compatible observation is that until 7–9 months of age, infants do not detect a tone in a broadband noise even when the noise spectrum does not include the frequency of the tone (Werner, 2007). This effect is not well-understood so far. Interestingly, however, newborns are able to match stimuli across modalities (auditory and visual) by their intensity (Lewkowicz and Turkewitz, 1980), a task on which adults perform quite poorly.

Based primarily on visual attention studies, it has been argued that between 2 and 4 months of age, infants' responses to stimuli becomes increasingly influenced by their longer-term previous experience and infants orient more toward novelty (Gomes et al., 2000) than to physical stimulus features. Then, at about 9 months of age, there is evidence for a reduction in the orienting response to novel visual stimuli (Ruff and Rothbart, 1996). However, because the maturation of the visual and auditory systems is not fully parallel (Anderson et al., 2001; Anderson and Thomason, 2013), the periods of characteristic changes in infantile responses may differ between the two modalities. In general, developmental specialization and narrowing of the initially broadly tuned perception of infants probably help reduce distraction by irrelevant stimuli (for a review, Gomes et al., 2000).

ERPs provide real time indices of information processing in the brain. The processing of acoustic deviance is usually studied in variants of the “oddball” paradigm: sequences based upon some regular relationship (“standard” stimuli) are infrequently interrupted by deviant stimuli violating the regularity. In the simplest and most commonly used oddball variant the standard is a repeating sound that is occasionally exchanged for a different (deviant) sound. Note, however, that also violations of quite complex regularities have been successfully tested in adults (e.g., Paavilainen et al., 2007) as well as in newborn infants (e.g., Ruusuvirta et al., 2009). The electrophysiological response to such deviations is assessed by subtracting the evoked potentials elicited by a control sound from that to deviant sound (for a discussion of how an appropriate control can be established, see Kujala et al., 2007). In adults, the subtraction between the ERPs to deviant and control sounds results in a negative difference waveform typically peaking between 100 and 200 ms from the onset of deviation, termed the Mismatch Negativity (MMN, Näätänen et al., 1978; for a recent review, see Näätänen et al., 2011). The currently most widely accepted interpretation of MMN elicitation suggests that it is triggered by finding mismatch between the incoming sound and the predictions drawn from generative representations of the auditory regularities detected from the preceding sound sequence (Winkler, 2007; Garrido et al., 2009). Recording deviance-elicited responses is a feasible way to assess neonatal auditory discrimination and regularity detection abilities, since it can be recorded even in the sleeping infants (Alho et al., 1990a).

In adults (Escera et al., 2000; Polich, 2007) and school-age children (Čeponienė et al., 2004; Gumenyuk et al., 2004), acoustically widely deviant stimuli (such as environmental sounds, often termed “novel” sounds in the literature, as well as sounds with wide spectrum or complex temporal structure delivered amongst pure or complex tones) usually elicit the frontocentrally positive P3a component peaking at about 300 ms from sound onset. Squires et al. (1975) proposed that the P3a was the central electrophysiological marker of the orienting response (see also Sokolov et al., 2002). The P3a is usually interpreted as a sign of attentional capture (Escera et al., 2000; Friedman et al., 2001), although recent evidence suggests that it may reflect processes evaluating the contextual relevance of deviant or rare sounds (Horváth et al., 2008).

## ERP components observed in the context of acoustic deviance detection and attention switching

### Neonatal ERP components indexing the detection and further processing of large acoustic deviance

In the majority of the studies testing acoustic deviance, a frontocentrally positive component peaking at about 300 ms was observed in newborn infants (e.g., Leppänen et al., 1997; Dehaene-Lambertz and Pena, 2001; Winkler et al., 2003; Novitski et al., 2007) and also in most fetuses (measured by magneto-encephalography; electric polarity not known; Draganova et al., 2005, 2007; Huotilainen et al., 2005). In contrast, a broad long-lasting later negativity (270–400 ms) was found in response to relatively modest auditory deviations both in newborns and in prematurely born infants (e.g., to the difference between Finnish vowels /y/ and /i/, Cheour-Luhtanen et al., 1995, 1996). Leppanen et al. (2004) suggested that the polarity of the deviance-detection response depends on the maturational level of the newborn, because immature neonates have been shown to display inverse polarity ERP responses (Kurtzberg and Vaughan, 1985). Yet other studies found earlier (<200 ms) and relatively narrower negative discriminative ERP responses in neonates (Alho et al., 1990a; Čeponienė et al., 2000, 2002a; Tanaka et al., 2001; Hirasawa et al., 2002; Kushnerenko et al., 2002a; Morr et al., 2002; Stefanics et al., 2007). Although it has been suggested that the polarity of the response may be related to the sleep stage or sleep vs. wakefulness (Friederici et al., 2002), this hypothesis has not been confirmed by the results of other studies (Leppänen et al., 1997; Cheour et al., 2002a; Hirasawa et al., 2002; Martynova et al., 2003). The earlier negativity and the later positivity have been observed together in some studies (Fellman et al., 2004; Kushnerenko et al., 2007; Háden et al., 2009) and compatible magnetic responses were observed by Sambeth et al. (2006). Kushnerenko et al. (2007) suggested that the early negativity was elicited by large spectral changes (e.g., 500 Hz tone vs. broadband noise). The above summary shows that the results obtained for ERPs elicited by acoustic deviance are quite diverse and it is not yet known, what variables (maturity, the degree of acoustic deviation, sleep-awake state, the length of the interstimulus interval, presentation rate, etc.) affect the morphology of the ERP responses (for a detailed discussion, see Háden, 2011).

**Figure 1:**
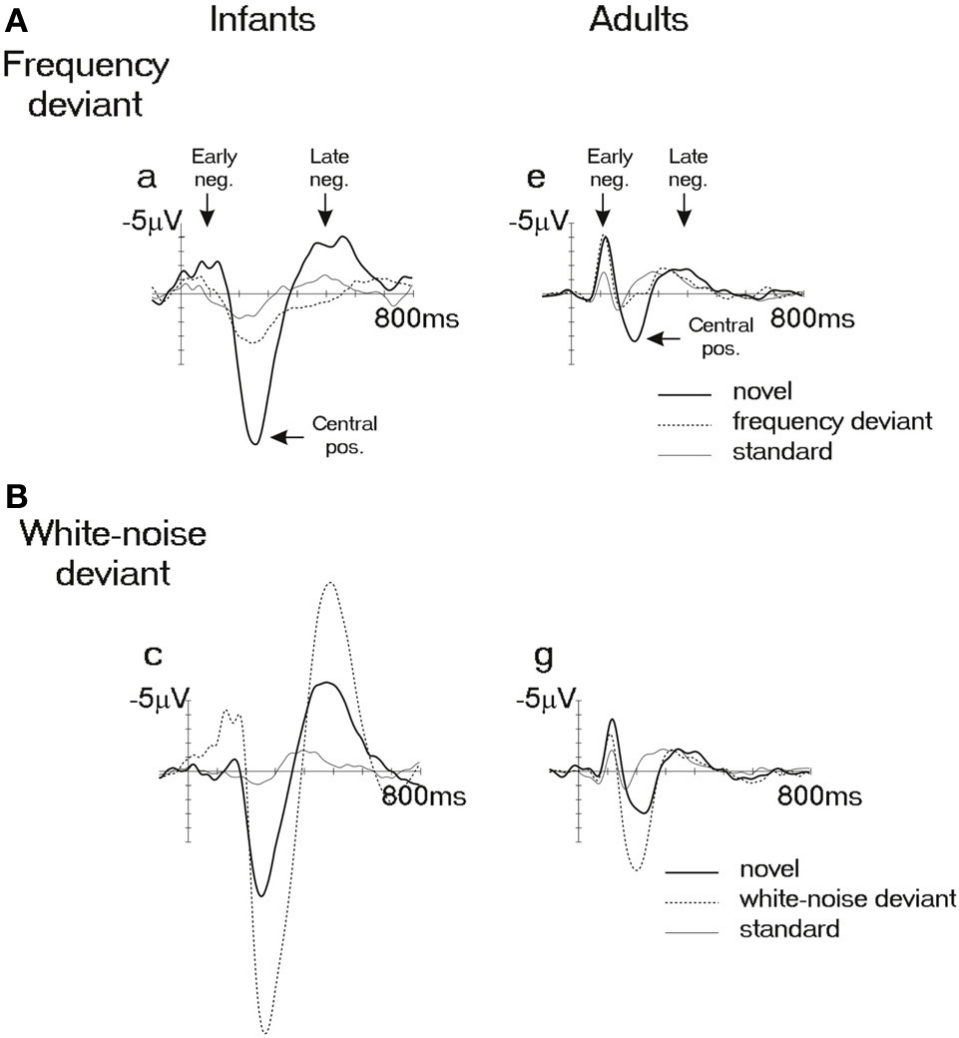
**Group-averaged ERPs elicited by harmonic tones and broadband sounds in newborns (*N* = 12; left side) and adults (*N* = 11; right side) in an oddball paradigm**. Upper row: the repetitive tone sequence (standard, thin continuous line) was occasionally broken by a higher-pitched tone (dotted line) or by various environmental (novel) sounds (thick continuous line). Bottom row: the repetitive tone sequence (standard, thin continuous line) was occasionally broken by white-noise segments (dotted line) or by various environmental (novel) sounds (thick continuous line). Observe the similar patterns of response to environmental sounds in newborns and adults by comparing the waveforms depicted by thick continuous lines on **(A** and **B)** with each other. The early negative peak is first followed by a central positive wave and then by a broad negativity. Stimulus onset is at the crossing of the two axes. Negative amplitudes are marked upwards. Adapted with permission from Kushnerenko et al. (2007).

Kushnerenko et al. (2007) have shown that broadband noise as well as “novel” sounds (diverse complex sounds, such as clicks, whistles, chirps, simulations of bird vocalizations) infrequently appearing amongst tones elicited high-amplitude ERPs in newborns. The responses consisted of an early negativity (EN) peaking between ca. 150–220 ms, followed by a large positivity (PC) at about 250–300 ms and a late negativity (LN) commencing at about 400 ms (see Figure 1, left panel). In contrast to many infant ERP studies reporting high levels of individual variability in very young infants, these stimuli reliably elicited all three components in all neonates. The requirement of large spectral deviation suggests incomplete maturation of frequency-specific pathways, and it is consistent with evidence showing that frequency resolution and fine frequency tuning is quite rough in neonates (Olsho et al., 1987, 1988; Novitski et al., 2007), improving rapidly during the first 6 months of life (Abdala and Folsom, 1995; Werner, 1996).

Surprisingly, newborns have shown a similar pattern of response to “novel” sounds and noise segments as was found in children and adults: the positive component (PC) resembles the auditory P3a (Kushnerenko et al., 2002a, 2007; see Figure 1, right panel). However, a major part of the PC in newborns is probably elicited by the spectrally rich “novel” sounds activating fresh afferent neurons (i.e., ones which did not respond to the frequently presented stimuli of the sequence). In adults, sound energy and spectral differences showed much smaller effects on ERP responses than in newborns, whereas contextual changes (e.g., a “novel” sound delivered amongst repetitive white-noise segments) were detected faster than in infants (Kushnerenko et al., 2007). Results of a new study aimed at separating contributions to the observed ERP waveforms associated with spectral deviance vs. contextual novelty (Háden et al., under review) suggest that whereas the neonatal response to noise segments is not modulated by the presence or absence of the surrounding tones (i.e., the acoustic context), the processing of environmental sounds is context-dependent. Therefore, it appears that the roots of the distinction between mere acoustic deviance and contextual information are already present at birth. Thus, despite the morphological similarity of the responses in Kushnerenko et al.'s (2007) study, the PC elicited by contextually novel sounds may show more similarity with the adult P3a than the response to noise segments. It should, however, be noted that maturation and learning still play a large role in refining and speeding up the processes separating acoustic deviation from contextually relevant information.

The PC in infants, similarly to the P3a in children, is sometimes followed by a late frontal negativity (LN) peaking between 500 and 600 ms latency in infants (Kurtzberg et al., 1984; Dehaene-Lambertz and Dehaene, 1994; Friederici et al., 2002; Kushnerenko et al., 2002a, 2007) and children (Čeponienė et al., 2004; Gumenyuk et al., 2004). This late negativity (LN) is larger in amplitude in younger than in older children (Bishop et al., 2010) showing the same maturational profile as has been previously reported for the negative component Nc (Courchesne, 1983). Nc has been suggested to reflect enhanced auditory (and visual) attention, as it was elicited in response to surprising, interesting, or important stimuli (Courchesne, 1978, 1990). A similar negativity was also found when participants had to re-orient their attention back to a task after distraction by ‘novel’ sounds (Escera et al., 2001) or in response to unexpected frequency changes in auditory stimuli (Schröger and Wolff, 1998; Schröger et al., 2000). This negativity was called the reorienting negativity (RON) by Schröger et al. (Schröger and Wolff, 1998). Being of comparable latency and scalp topography, the Nc and RON might, in fact, reflect the same neural process. The elicitation of the Nc-like LN component in neonates suggests that, perhaps, they may also able to return to a previous context following the processing of a salient stimulus.

In summary, although the results of neonatal ERP studies are far from being unequivocal, some evidence suggests that the main elements of the chain of distraction-related processes are already present at birth. At least from birth onwards infants detect auditory deviance. High amounts of acoustic deviance elicit large ERP responses. Infants may also differentially process sounds with only large acoustic deviance from contextual novelty, showing to the latter a response resembling those associated with attention switching (or contextual evaluation) in adults. Finally, they may also show signs that after the processing of acoustic novelty the original context may be re-established. Note that this summary is highly speculative and is mainly included for provoking future testing of these ideas.

### Development of ERP components indexing the detection and further processing of large acoustic deviance between 2 and 6 months of age

During first 6 months of life, auditory ERP amplitudes dramatically change in absolute voltage level as well as in relation to each other (see, Kushnerenko et al., 2002b; Kushnerenko, 2003; Jing and Benasich, 2006; Csibra et al., 2009). The development of the ERP responses related to auditory deviance detection generally follow the maturational pattern of the obligatory ERP responses (i.e., responses also elicited by the regular auditory stimuli, the standards; Kushnerenko et al., 2002a,b). Kushnerenko et al. (2002a,b) employed a paradigm allowing the separation of the contributions of auditory deviance detection and differential refractoriness to the ERP responses elicited by deviant sounds. The latter is caused by standards being delivered more often than deviants and thus eliciting a lower-amplitude response than what the same sound would elicit if delivered as infrequently as the deviant. Thus, when the ERPs of the standard and the deviant are compared, the difference between them sums together the contribution from the neural processes of deviance detection and that related to the shorter average ISI between successive standards compared with successive deviants (for a detailed discussion, see Kujala et al., 2007). Therefore, in addition to the oddball stimulus block, Kushnerenko et al. (2002a,b) administered also a separate (control) stimulus block in which the “deviant” tone from the oddball stimulus block was delivered equiprobably together with two other tones. For assessing the genuine deviance-detection related neural response, they then compared the response elicited by the deviant from the oddball sequence with that elicited by the same tone in the control stimulus block (termed the “control” tone). Jacobsen et al. (2003) have shown that this procedure provides a good estimate of the genuine deviance-detection related contribution to the deviant-stimulus response. Kushnerenko et al. (2002a,b) found that the amplitude of infantile P2/PC increased almost 3-fold from birth to 3 months of age both in response to the deviant and the control sounds. Between 3 and 6 months of age, the ERP waveforms become better defined with the broader deflection gradually giving way to sharper peaks. As part of this general development of the ERP responses, the P2/PC merges into the P150-N250-P350 complex, which is then followed by the N450 wave. In parallel, by 6 months of age, the pattern of the auditory deviance detection response as derived by subtracting the control ERP from that elicited by the deviant, can be described in terms of the sequence of EN-PC-LN (with peaks appearing at 200, 300, and 450 ms, respectively). Kushnerenko et al. (2002a,b) delivered complex tones and they employed pitch deviance of 50% (harmonic tones, 500 Hz standard, 750 Hz deviant). Despite the large deviation, these stimuli only elicited reliable EN-PC-LN responses at the age of 6 months, but not before. In contrast, as was described in the previous section, white-noise segments (presented in the context of the same complex tones) elicited all three components already at birth (Kushnerenko et al., 2007).

For the ERP responses elicited by deviance carried by spectrally rich sounds (piano tones), the broad discriminative positivity turns into EN followed by PC between 2 and 4 months of age (He et al., 2009). Further, with increasing pitch separation (from 1/12 octave to ½ octave), the amplitude of the EN/PC increases while its latency decreases. No similar magnitude of deviance effects were observed for the broad positivity found in 2-month olds. Thus, the component structure that have been observed in response to white-noise segments at birth emerges later in response to pitch changes. This suggests that the resolution of pitch representation improves between 2 and 4 months, earlier than the estimate based on behavioral studies (Olsho et al., 1982; Abdala and Folsom, 1995; Werner, 1996).

**Figure 2:**
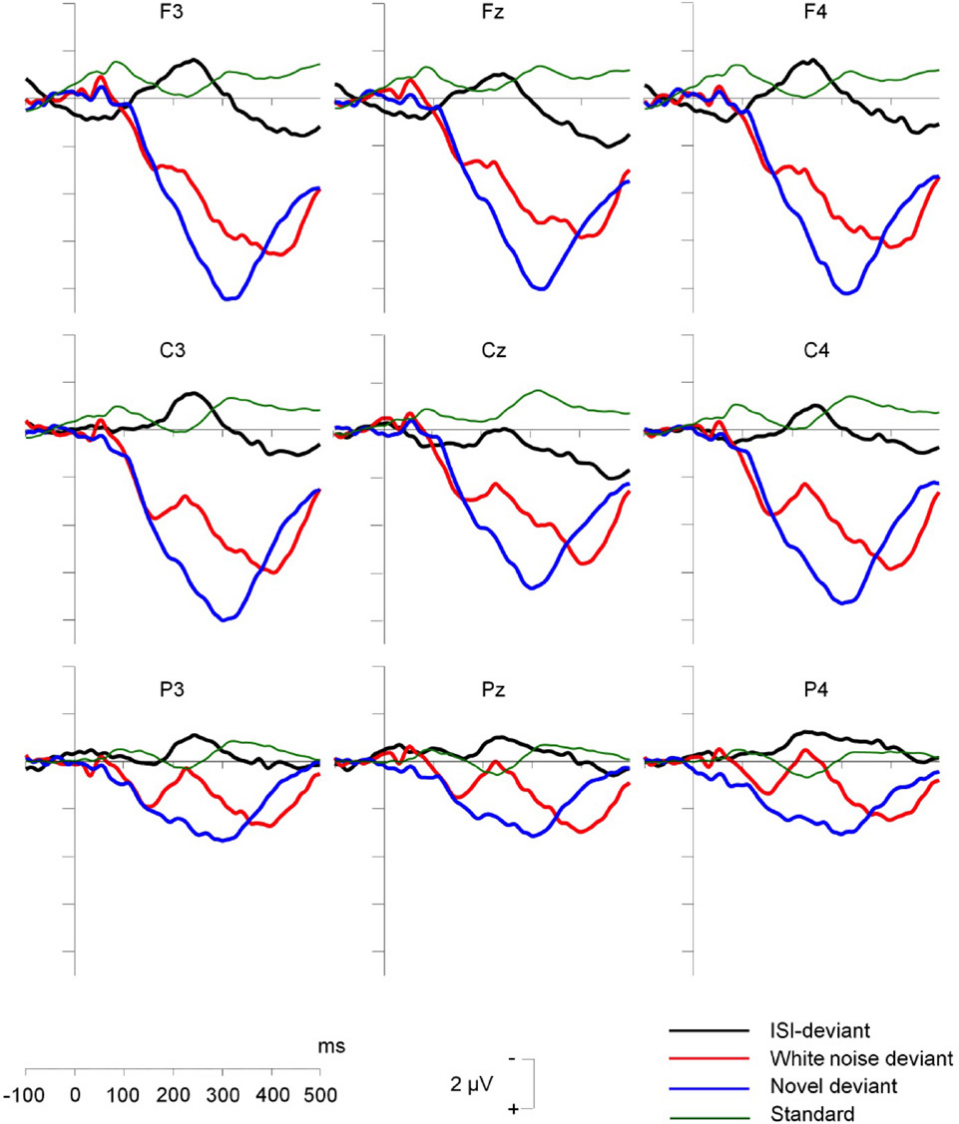
**Group-averaged (*N* = 36 waking 2 months old infants) difference waveforms for tones delivered occasionally too early in an otherwise isochronous tone sequence (ISI-deviant; black line), rare white noise segments (red line) and novel sounds (blue line); the ERP response to the frequent regular tone (standard, shown on the figure by green line) has been subtracted from each of the other ERP responses**. Stimulus onset is at the crossing of the two axes. Negative amplitudes are marked upwards. Amplitude calibration is at the bottom of the figure. Reprinted from Otte et al. (2013).

As was reviewed in the previous section, rare noise and environmental sounds presented amongst frequent tones elicited morphologically similar ERPs responses in newborn infants (Kushnerenko et al., 2007). By ca. 2 months of age, morphological differences emerge between the ERPs elicited by rare noise and environmental sounds. Otte et al. (2013) adapted the oddball paradigm of Kushnerenko et al. (2007) and studied a relatively large group of 2 month old infants. Embedded in a regular sequence of a repetitive complex tone (500 Hz), these authors presented a temporal (interstimulus interval) deviant, as well as rare environmental (dog barking, doorbell ringing, etc.) and white-noise sounds. Visual inspection of the ERPs (Figure 2) of Otte et al. (2013) suggest that whereas the white noise segments elicited a response pattern that was similar to the typical P150-N250-P350 sequence of deflections (especially in the waking infants), the environmental sounds elicited a P3a-like prolonged positive response in both waking and sleeping infants. Although Otte et al. (2013) exercised caution in interpreting this large positive response as a precursor of the adult P3a, Háden et al.'s (under review) observations regarding differences in contextual processing of noise and environmental sounds in newborns increase the plausibility of their speculative interpretation. Van den Heuvel et al. (under review) using the same paradigm as Otte et al. (2013) showed that at 4 months of age the distinction between ERP response to novelty vs. white noise was more clear and that frontal activation in response to unique environmental sounds was increased ad compared with 2-month-old infants.

The emerging structural differences in the ERP responses elicited by rare sounds with similar large acoustical deviance but different amounts of contextual information are compatible with behavioral observations suggesting that between birth and 4 month, the role of contextual novelty in triggering orientation toward incoming stimuli increases at the expense of physical stimulus features (Ruff and Rothbart, 1996). While Háden et al. (under review) results suggest that newborns process environmental, but not noise sounds differently depending on the context they appear in, both of these sounds may result in similar brain activation when encountered as deviants within the same context. In contrast, the observations from Otte et al.'s (2013) results show the first indication in 2-month olds that these two types of sounds are processed differently even within the same context. He et al. (2009) results demonstrated further development of the responses between 2 and 4 month and, finally, Kushnerenko et al.'s (2002a,b) data suggest that these response patterns stabilize and extend to somewhat more subtle types of deviations by the 6th month of life.

### Development of ERP components indexing the detection and further processing of large acoustic deviance between 6 and 12 months of age

During second half of the first year of life, the amplitude of the PC to large spectral deviations decreases (Kushnerenko et al., 2002a; Morr et al., 2002). In their longitudinal study testing infants at 3, 6, 9, 12, and 24 months of age, Kushnerenko et al. (2002a) found that the PC (termed there as discriminative positivity, DP) elicited by 50% pitch separation between frequent (500 Hz) and infrequent (750 Hz) harmonic tones reached its maximal amplitude at 6 month of age (~10 μV) decreasing thereafter by 9 months (~4–6 μV) and further at 12 months of age (~3 μV). Compatible results were obtained by Morr et al. (2002), who measured ERP responses in infants for both small (20%; 1000 vs. 1200 Hz) and large pitch deviations (100%; 1000 vs. 2000 Hz) carried by pure tones. These authors found that the PC in response to a large pitch change (100%) was largest between 3 and 7 months of age (~5 μV), and decreased gradually thereafter: by the age of 13–18 months the peak amplitude of the PC was ca. 3 μV and by the second year it almost completely disappeared and a negative discriminative response more similar to the child/adult MMN (Csépe, 1995; Shafer et al., 2000; Wetzel et al., 2006) emerged as the primary response to acoustic deviance.

**Figure 3:**
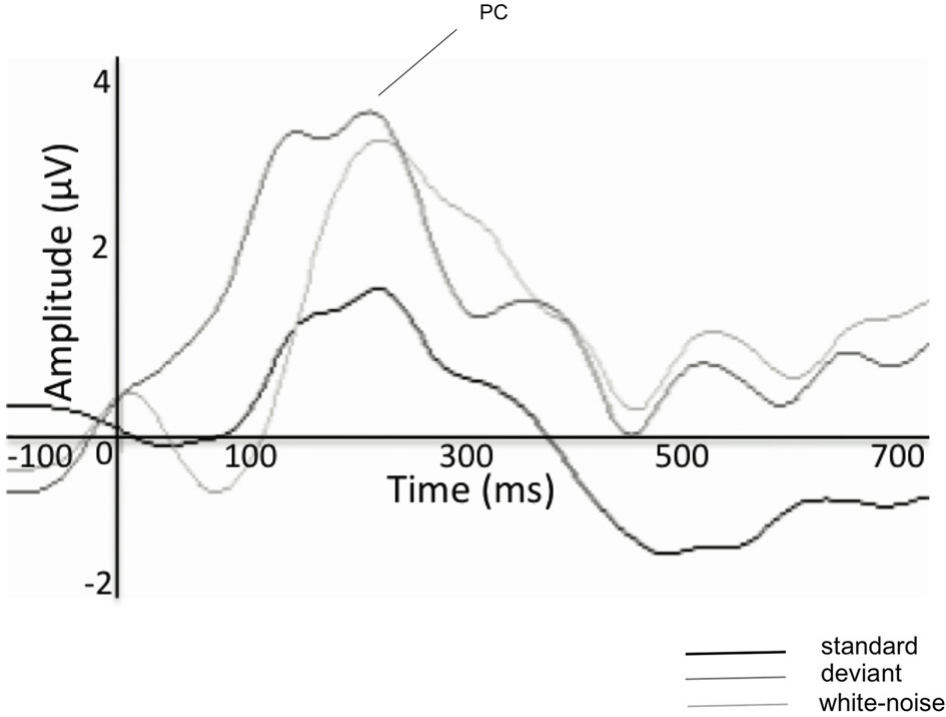
**Group-averaged (*N* = 21 9 months old infants) ERPs elicited by the frequent repeating tones (standards, black line), pitch deviants (dark-gray line), and noise segments (light-gray line)**. In Guiraud et al. (2011) the P2/PC peaked at about 200 ms and it is denoted as P150. Stimulus onset is at the crossing of the two axes. Negative amplitudes are marked downwards. Adapted with permission from Guiraud et al. (2011). Promotional and commercial use of the material in print, digital, or mobile device format is prohibited without the permission from the publisher Lippincott Williams & Wilkins. Please contact journalpermissions@lww.com for further information.

During the same period, the difference between the response amplitudes elicited by larger and smaller acoustic deviation decreases as compared to that in younger infants. Whereas at birth, the amplitude of the P2/PC to infrequent white noise segments delivered amongst frequent tones was almost three times as large as the PC elicited by pitch deviance between two tones [see Figure 1, adapted from Kushnerenko et al. (2007)], at 9 months of age the amplitude difference between the white-noise and frequency deviants was much less [see Figure 3, adapted from Guiraud et al. (2011)], who presented the stimulus paradigm adapted from (Kushnerenko et al., 2007) to 9 months old infants).

However, the PC amplitude in response to rare environmental sounds does not decrease during the same period. Marshall et al. (2009), showed that at the age of 9 months infants still show large (~10 μV) PC responses to rare environmental sounds. Kushnerenko et al. (2002a) compared the responses elicited by large pitch deviation (50% pitch separation between frequent and infrequent complex tones, 500 vs. 750 Hz) and rare unique environmental sounds in 2-year olds. While the PC to pitch deviance had a lower amplitude (~4 μV) compared with younger infants, the PC elicited by contextual novelty was large (~9 μV) and showed a second frontally dominant peak, similar to the P3a observed in school-age children (Gumenyuk et al., 2001; Wetzel and Schröger, 2007; Wetzel et al., 2009).

In summary, whereas the effects of the magnitude of acoustic deviation on the PC component diminishes from ca. 6 months of age, and, in general, the PC response to acoustic deviations gradually decreases from this age onward, when acoustic deviation is coupled with contextual novelty, the PC response stays large. Finally, at about 2 years of age, the PC to acoustic deviation is exchanged for a negative discriminative response (the MMN), whereas the PC elicited by contextual novelty is transformed into the P3a response. However, it is not yet possible to establish an exact timeline of the development of the PC response or the amplitude norms as a function of age, because different studies presented different stimuli and interstimulus intervals, as well as analysing the ERPs with different filter settings. For example, applying a high-pass filter with 1 Hz or higher cut-off frequency is known to substantially attenuate the amplitude of a slow waves, such as the PC or P3a (Holinger et al., 2000; Luck, 2005). Further, the PC amplitude has been shown to also depend on the stimulus presentation rate (He et al., 2009).

The maturational changes of the PC component amplitude presumably reflect changes in the sensitivity of the infant brain for orienting toward different aspects of auditory information. Sensitivity to acoustic deviance appears to be heightened during the infancy period compared with children and adults, as unlike in older children and adults, the PC is elicited also in response to small acoustic deviations. In school-age children and adults, the P3a component is elicited almost exclusively by large acoustic deviance and/or contextual novelty. One may speculate that the decrease of the PC amplitude to acoustic deviance (but not to contextual novelty) during the first year of life may reflect the development of a network in the infant brain suppressing the processing of contextually less informative stimuli. Interestingly, this is also true for audiovisual integration studies in infants: similar to PC central right positivity in response to mismatch between lips movement and speech sound decreased in amplitude by about 9 months of age, presumably assimilating it to the closest possible sound (Kushnerenko et al., 2008, 2013a,b). However, one should be cautious with this interpretation, as the PC response may also sum contributions of processes not related to orienting (or in terms of ERPs, neural circuits not involved in the generation of the P3a in older children and adults).

## Discussion

Delivering acoustically widely deviant and/or contextually novel sounds have been suggested as a good model for studying passive attention, distraction, and reorientation (e.g., Escera et al., 2000). We argued in the introduction that the measurement of brain activity provides opportunities for following the development of these functions during infancy which cannot be achieved by behavioral methods. Of the various brain measures, EEG and the analysis of ERPs is by far the most easily accessible and widely used research method. Further, electromagnetic brain responses to acoustically widely deviant and contextually novel sounds have been well-studied in adults (Escera et al., 2000; Friedman et al., 2001; Polich, 2007) and to some degree in children (see the papers appearing in the current Research Topic). Thus, employing these methods in young infants allows one to interpret results in the context of development. We conclude our review by discussing (1) the development of the ERP components during the first year of life; (2) methodological issues regarding ERPs observed in the deviance/novelty paradigm in infants; (3) possible application of these methods in clinical and at-risk groups; and (4) directions for future research.

### Overview of the development of ERP components indexing the detection and further processing of large acoustic deviance during the first year of life

In the previous sections, we reviewed evidence compatible with the notion that ERP components similar in some characteristics to those observed for the cycle of deviance detection, distraction, and reorientation in adults (the MMN, P3a, and perhaps even RON) can be recorded very early during infancy. We then followed the development of these components during the first year of live, also hinting at their further transformation into the component structure observed in pre-school age children. It is obvious that there are many marked differences between adults and infants both in processing auditory information (probably sensory information, in general) and perhaps even greater differences in attentional functions. For example, it is assumed that because of the weak axonal myelination and less efficient cortical networks, the infant brain is relatively slow in processing auditory information when compared to the adult brain (Dehaene-Lambertz et al., 2010). Thus, by no means do we wish to suggest that either the possible infantile precursors of the adult ERP responses or the underlying cognitive processes in infants are full analogues of their adult counterparts. However, given that orienting toward new information is likely an innate function manifesting very early during infancy, it may not be counterintuitive to suggest that at least rudimentary forms of each aspect of passive-attention/distraction are functional and quickly developing in early infancy.

In a recent review on infant speech perception and the development of the linguistic brain network, Dehaene-Lambertz (2011) concluded that in the last decades, neuroimaging has completely changed the previously prevailing view of the infant brain as “a few islands of functional cortex amongst a vast space of barely functional immature regions” (p. 185). NIRS, fMRI and diffusion tension MRI (DTI) have enabled the *in vivo* study of the maturation of brain structures. For example, the mapping of the spatial organization of white matter in fiber bundles with DTI (Mori and Van Zijl, 2002), revealed that arrangement, density, and myelination of the fibers change with age and at different rates across bundles (Neil et al., 2002; Dubois et al., 2006). As Dehaene-Lambertz (2011) concludes, “a structured [brain] organization is present from the first days on” and that ‘this initial architecture has been selected through human evolution as the most efficient to help infants to pick the correct cues in the environment in order to build the rich and large social groups seen in humans’ (p. 196). In a similar vein, several studies showed that despite the low resolution of auditory features in neonates, some higher-level auditory perceptual mechanisms are already functional in newborn infants; e.g., auditory stream segregation (Winkler et al., 2003), extracting invariant higher-order auditory features from a variable input (Carral et al., 2005; Háden et al., 2009; Ruusuvirta et al., 2009), grouping mechanisms (Stefanics et al., 2007), even the formation of hierarchical representations (Winkler et al., 2009), and discrimination of speech sounds from other types of sounds (Vouloumanos and Werker, 2007). Thus, the initial brain organization enables processes that are crucial for the further development of cognitive abilities. As the above listed functions had ERP footprints, one may speculate that the processes of passive attention/distraction may also be observed through ERPs. This motivates the hypothesis to be tested by future research that the infantile EN, P2/PC, and LN/NC include precursors of the adult MMN, P3a, and RON.

It is, however, important to qualify the above hypothesis. The infantile ERP waveforms listed above do not show a one-to-one correspondence to the adult counterparts. This is clear from results showing that, in addition to being sensitive to deviance, the EN is modulated by spectral richness while PC is modulated by sound intensity (Kushnerenko et al., 2007). Thus, whereas both of these responses may reflect the functioning of auditory deviance detection (including higher-order, “abstract” deviations) and therefore can be suggested as precursors of the adult MMN, they also show characteristics which are typical for the ERP correlates of auditory feature extraction in adults (such as the auditory P1 and N1; see, e.g., Näätänen and Winkler, 1999). Currently, it is not possible to decide, whether the mixture of the characteristics of infantile ERP waveforms (compared with the finer specialization found for the adult ERP components) indicate that the underlying processes are initially not fully separated or that this is a problem of the sensitivity of ERP measures in young infants. If the former was true, these observations then provide important insights into the early development of cognitive functions. In any case, while the infantile ERPs related to processing of large acoustic deviance and contextual novelty may show some of the features characterizing the chain of adult ERP responses observed during distraction by sounds, both the underlying functions and their ERP correlates undergo a long maturational period before reaching the form observed in adults.

Finally, it is of interest to compare the development of attention to salient visual stimuli with those in the auditory modality. In a recent article Leppänen et al. (2011) describe how during the first months of life diverse fundamental components of spatial attention are acquired that allow infants to engage, disengage, and shift visual attention in a flexible way. While between 1 and 3 months of age, terminating the processing of a stimulus in the attentional focus and orienting to a new stimulus, is difficult for infants (Hood, 1995), between 2 and 4 months this becomes easier, and by the age of 6 months infants reach the ability to flexibly disengage attention from its current focus and shift to a new stimulus in another spatial location (Posner and Petersen, 1990; Hunnius et al., 2006; Hunnius, 2007). Recent research has demonstrated that by 6–7 months of age, affective significance starts to influence the infants' visual attention (Peltola et al., 2008; Leppänen et al., 2010, 2011) which is reflected in the relatively greater difficulty in disengaging attention from affectively salient facial expressions. A similar trend was observed in audiovisual speech integration studies (Tomalski et al., 2012; Kushnerenko et al., 2013a,b): between 6 and 9 months of age, there is a shift of visual attention from familiar combinations of articulatory lip movements and sounds to the novel combinations. The hypothesis that early-developing attentional control processes play a vital role in the development of more advanced cognitive and emotional skills is an important one. The efficiency of attention disengagement and other aspects of attention regulation in infancy, is not only linked with cognitive and emotional functioning in infancy and later childhood (Johnson et al., 1991; Frick et al., 1999; Fox et al., 2008) but also with language development (Kushnerenko et al., 2013b). Although the traditions of experimental design are somewhat different between the two fields, preventing any direct comparison between them, it is clear that there are parallels in development. Separation of physical and contextual deviance goes hand in hand with the shift from rigid focusing to more flexible disengagement and then to the increased relevance of social/emotional information. One important direction of future research is to delineate the development of the common attentional processes and resources from modality-specific phenomena. This will require the development of equivalent and multimodal stimulus paradigms.

### Methodological issues regarding the use of ERP method for studying passive attention in young infants

There are a number of methodological and interpretational problems relevant to the deviance/novelty paradigm. ERP responses often overlap each other and thus the recorded waves do not have a one-to-one correspondence to specific processes. Näätänen and Picton's (1987) defined an ERP component as follows: “… we define an ERP ‘component’ as the contribution to the recorded waveform of a particular generator process, such as the activation of a localized area of cerebral cortex by a specific pattern of input” (p. 376). However, these criteria are seldom met even by ERP responses recorded in adults despite the far better signal to noise ratios achievable and the better tools available for source localization (e.g., possibility to run structural MRI to set up a realistic head model) than what is possible in young infants. While advances in source localization and the use of converging imaging methods may gradually help to alleviate the problem of separating contributions from generators located in closely spaced brain areas, a lot of work remains to be done for finding the experimental variables that affect the various ERP responses in young infants. There are a number of variables, whose effects have not been systematically tested in any age group and even those few studies, which have assessed some parametric effect often recorded rather small groups of infants. Thus, these results may need to be reconfirmed. Regarding the deviance/novelty paradigm, it is well-known from studies in adults that the ERP waveforms elicited by large deviance sum together contributions from generators sensitive to at least three different aspects of the stimuli: (1) neuronal populations sensitive to some stimulus feature present in the deviant but not in the standard (e.g., if the frequent and the infrequent stimuli are tones differing in frequency, they activate partly different neuronal populations in the tonotopically organized parts of the auditory system); (2) differential refractoriness or adaptation of the neurons activated by the frequent and the infrequent stimulus (e.g., the neurons only activated by the deviant, but not by the standard are activated quite less frequently and, therefore, may respond more vigorously than those activated by both stimuli); and (3) activation resulting from auditory mismatch (neurons activated by the difference between the incoming sound and the one predicted on the basis of the preceding sounds—the “error” signal in terms of predictive coding theories; (Winkler, 2007; Garrido et al., 2009). Contributions to the observable ERP response by neurons sensitive to contextual mismatch, calling for further processing, and possibly reorientation may come on top of these. Based on the congruent results of a very large number of ERP studies in adults, researchers have a reasonably good idea about which of these variable may cause temporally overlapping ERP responses. As a result, there are guidelines and paradigms for extracting one or another of these responses from the summed ERP waveforms (e.g., Kujala et al., 2007, provide suggestions for separating the response to auditory mismatch from the stimulus-specific responses and differential refractoriness in the auditory oddball paradigm). However, some of the assumptions for adults may not hold for young infants (or even change within a few months during early development). Further, as was mentioned in Section Neonatal ERP components indexing the detection and further processing of large acoustic deviance, variables not specifically related to the deviance/novelty paradigm may also affect the ERP responses recorded in it (for a review, see Háden, 2011). And, finally, to interpret the associations between the (presumed) neural changes and ERP components one needs also to take into account the age-related changes at the macro structural level. Indeed, changes in physical features of the brain (such as thickness of the skull, bone conductance, relative position of sulci and gyri) may influence the recording of electrical activity from the scalp (Luck, 2005; De Haan, 2007).

To date there is not enough evidence to establish to what extent head size and shape and skull thickness affect ERP amplitudes and latencies. A few studies have reported significant negative correlations between P3 amplitude and scull thickness in adult participants (Pfefferbaum and Rosenbloom, 1987; Frodl et al., 2001). Individual differences in P3 amplitudes varied from 10–15 μV for ~5–6 mm scull to 4–6 μV for 7–10 mm skull (Frodl et al., 2001). There is also data indicating that in infants with deformational posterior plagiocephaly, the amplitude of the auditory P150/N250 complex is less than half the size of that observed in control infants (−1.91 μV vs. −5.11 μV for N250) (Balan et al., 2002).

Thus in order to allow more specific interpretation of the ERP responses observed in young infants, many more studies are needed to test the effects of various stimulus parameters, age-related structural changes, and to set up procedures to separate temporally overlapping contributions to the recorded waveforms. As an example, the latency decrease from birth to 6 months of age of the infantile P2 is probably mostly due to the emergence of the N250, which divides the P2 into two peaks, one at ca. 150 and another at ca. 350 ms (Kushnerenko et al., 2002b).

### Studying clinical and at-risk groups

The Developmental Origins of Behavior, Health and Disease (DOBHaD; Van den Bergh, 2011) hypothesis addresses the short- and long-term consequences of the conditions of the developmental environment for phenotypic variations in behavior, health and disease (Barker, 1998; Gluckman and Hanson, 2004; Seckl and Holmes, 2007; Plagemann, 2012). Recording ERPs in young infants provides important tools for this approach to development. Since large acoustic deviance evokes reliable ERP responses in individual infants from birth onwards (Kushnerenko et al., 2007), it may be better suited for studying clinical and at-risk groups than small acoustic deviance, the responses to which substantially vary even across typically developing infants (Kurtzberg et al., 1984; Kushnerenko et al., 2002a). Marshall et al. (2009) suggested that the high individual variability in processing small and moderate amounts of acoustic deviance may be partly explained by temperamental differences, which in turn may be linked to differences in early sensory processing.

Marshall et al. (2009) demonstrated that while there were no significant differences in processing novel (environmental) sounds between infants sorted by behavioral testing into high-positive and high-negative temperamental groups, the infants in the high-negative group showed increased attentional engagement (larger PC) to a small acoustic deviation that was neither salient nor contextually novel. One possibility is that temperamental differences are related to properties of the sensory pathway (Galbraith, 2001; Bar-Haim, 2002). Galbraith (2001) suggested that selective filtering of irrelevant information might be modulated at the level of the auditory nerve while Bar-Haim (2002) argued that differences in the sensitivity and functioning of peripheral neuronal processes may contribute to individual differences in introversion and social withdrawal with greater incidence of abnormal middle ear acoustic reflexes and faster ABR latencies for introverts than extraverts. Although the relative roles of top-down and bottom-up processes cannot be assessed from the results of Marshall et al. (2009), their ERP evidence corroborates previous behavioral data demonstrating that auditory orienting in newborns can be predictive of temperamental traits later in infancy (Riese, 1998). Thus, it appears that heightened sensitivity/reactivity in neonates to auditory information that is neither novel nor relevant may be indicative of the future development of some temperamental traits.

Increased sensitivity to irrelevant auditory information is of particular interest for studying infants at-risk for developing attentional or social problems (e.g., autism spectrum disorder (ASD), attention deficit-hyperactivity disorder (ADHD), social withdrawal, etc.). Sensory-perceptual abnormalities are present in about 90% of individuals with autism, including auditory hypersensitivity (Gomes et al., 2008). Guiraud et al. (2011) found that infants with high familial risk for ASD showed poorer ability to suppress a response to regular (low novelty) irrelevant sounds than control infants. That is, in response to the repeatedly presented standard sound, the infants in the low-risk group showed habituation of the P2/PC amplitude already by the second identical tone, whereas the high-risk group did not show a decrement of the same ERP response. As a result, in the latter group, no increase of the ERP amplitude was observed in response to small acoustic deviation. This suggests that high-risk infants may over-process regular (uninformative) sounds, which would otherwise not require limited capacities and, as a result, may have difficulties to selectively attend to more informative auditory stimuli, such as human speech. In school-age children, it was found that while the response to non-speech acoustic deviance was increased in individuals with ASD (Čeponienė et al., 2003; Ferri et al., 2003; Lepistö et al., 2005), the orienting to changes in speech sounds was impaired (as reflected by the diminished novelty P3a) suggesting difficulties in social orienting (Čeponienė et al., 2003; Lepistö et al., 2005).

Therefore, in addition to (but not incompatibly with) the peripheral sensitivity explanation for increased ERP amplitudes found in response to uninformative sounds in some at-risk groups, these studies suggest poorer ability to suppress brain responses to irrelevant sounds, which may be related to delayed/atypical maturation of inhibitory networks involved in sound processing. As we have summarized in Section Overview of the development of ERP components indexing the detection and further processing of large acoustic deviance during the first year of life, the amplitude of the PC decreases during the second half of the first year, which is the time when cortical layers IV, V and VI develop their connections (Moore and Guan, 2001; Moore, 2002). According to Moore and Guan (2001), prior to 4 months of age, only layer I of the auditory cortex is mature, but after the 4th month of life, the number of immunopositive axons in this layer is greatly reduced. The decrease of PC and development of the negative scalp-recorded components after 4 months of age could be related to these maturational changes and might be associated with development of intracortical inhibitory connections. Animal studies showed a drastic change in the relative weight of inhibitory but not excitatory synapses in the cortex during this period (Zhang et al., 2011).

The inhibition hypothesis receives further support from data obtained from preterm infants. High-amplitude PC responses (~10 μV) were observed to large acoustic but low-novelty deviance in two groups of prematurely born infants (one appropriate and the other small for their gestational age) at the age of 12 month (Fellman et al., 2004). In contrast, in the corresponding age-matched control groups, the PC was of less than 1 μV amplitude; rather, in the control groups, the response to acoustic deviance was characterized by the EN/LN complex, as is typical for older children (Čeponienė et al., 2002b, 2004). Alho et al.'s (1990b) results obtained in 4 month old preterm infants in response to a small acoustic deviation are compatible with Fellman et al.'s (2004) findings. It is, however, not known whether these atypical brain responses to auditory deviance result from preterm birth *per se* or also from the atypical early auditory experiences in the neonatal intensive care unit (Gray and Philbin, 2004; Brown, 2009; Lickliter, 2011).

Research in the past 15 years showed associations between prenatal exposure to high maternal anxiety/stress and behavioral problems, such as high negative reactivity, irritability, ADHD symptomatology, and delayed language problems (for reviews, see (Huizink et al., 2004; Van den Bergh et al., 2005; Räikkönen et al., 2011). In groups of neonates prenatally exposed to elevated levels of pregnancy-specific anxiety (DiPietro et al., 2010) or postpartum maternal anxiety (Harvison et al., 2009), altered brainstem (DiPietro et al., 2010) and auditory ERP responses (Harvison et al., 2009) were found. Results of a recent study delivering environmental sounds and noise segments amongst frequent tones (for the description of the paradigm, see Otte et al., 2013) suggest that 2-month-old infants prenatally exposed to high maternal anxiety show enhanced ERP responses to sounds with low information content compared to infants born to mothers with lower levels of anxiety (Van den Bergh et al., 2012, 2013; Van den Heuvel et al. under review). Similarly to infants at risk for ASD, allocating more than normal amounts of processing capacities to uninformative sounds may cause difficulties in extracting important auditory features, such as phonetic and prosodic cues. This in turn may lead to cognitive (language), emotional, and learning problems later in life, as has been found for children prenatally exposed to high levels of maternal anxiety (Laplante et al., 2004; King and Laplante, 2005; Talge et al., 2007; Laplante et al., 2008; Räikkönen et al., 2011; Loomans et al., 2012).

In summary, the ERP responses to small and large acoustic deviations in young infants may prove to be indicative not only of individual differences in reacting to novelty, but also of potential problems with sensory-cognitive information processing, which may then affect the ability to select information to attend and react to. The lack of selectivity to informative sounds may be detrimental for the development of selective attention and language acquisition, such as may be the case for infants at risk for ASD or prenatally exposed to high levels of maternal anxiety. Inability to suppress irrelevant information may also develop into problems with regulation of attention, which along with hyperactivity is often observed in prematurely born children (Aylward, 2002; Mick et al., 2002; Bayless and Stevenson, 2007; for a review, see Van de Weijer-Bergsma et al., 2008).

### Research goals: the three approaches of developmental cognitive neuroscience

The past two decades saw the emergence of developmental cognitive neuroscience (DCN), an interdisciplinary field aiming to relate cognitive development, including that of sensory, perceptual, and motor abilities to changes in the micro- and macro-structure of the nervous system and to genetic and epigenetic factors. Johnson (2010) described the following three, distinct but not necessarily incompatible viewpoints on human “functional brain development”: (a) a maturational perspective, (b) interactive specialization, and (c) skill learning. In what follows, we describe how the components of passive auditory attention (i.e., stimulus detection, distraction, and re-orienting) have been viewed in terms of these three approaches, also commenting on possible future developments.

#### The maturational perspective

According to this view, the research goal is to relate newly emerging sensory, perceptual/cognitive, and motor functions to (different aspects of) the maturation of particular brain regions or circuitry. Applied to the ERP research reviewed above, this has led to questions asking to what extent could the observed age-related differences, such as changes in the latency, peak amplitude, or scalp distribution of the EN, PC, and LN components during the first year of life be attributed to “maturation.” A typical example is to link the general shortening of ERP peak latencies to the increasing myelination of axons in specific brain regions. For example, the latency of visual ERPs evoked by flash stimuli decreased in a stepwise manner (about 6 ms/week) with an increased speed of latency decrease close to the 37th week from conception, which coincides with the onset of myelination of the optic radiation. In full-term infants, the P1 visual evoked potential decreases in latency from ~247 ms to 108 ms during next 19 weeks of life (~7ms/week) (McCulloch, 2007). These rates are different though for different components. For example, the latency of auditory N250 stays at about the same value from birth to preschool age, while the N450 latency decreases from 600 ms to 400 ms during the first year of life (~4ms/week) (Kushnerenko et al., 2002b). For MMN in infants and children a rate of 1ms/month latency decrease has been observed (Morr et al., 2002).

However, as was already discussed in Section Methodological issues regarding the use of ERP method for studying passive attention in young infants, without detailed knowledge about possible overlapping ERP components, the myelination explanation could be misleading. Further, results of some new studies suggest that the microstructural organization may be an important factor for the ERP peak latencies besides the general process of myelination (De Zeeuw et al., 2012; Chen et al., 2013). Johnson (2010, p. 14), critically remarks that “associations between neural and cognitive changes based on ‘age of onset’ can be theoretically weak due to the great variety of neuroanatomical and neurochemical measures that changes at different time in different regions of the brain.” Indeed, we saw that ERP measures suggest earlier improvement of pitch resolution than estimates based on behavioral assessment. The problem here is 2-fold: (1) is the emergence of an ERP response a valid indicator of the emergence of the corresponding sensory/cognitive advancement in the absence of behavioral confirmation and (2) ERP measures reflect the effects of a large number of changes in the brain, which, at least at this point cannot be specified. ERP amplitudes have been linked with the synaptic density in different cortical areas (Huttenlocher, 1979; Courchesne, 1990). For example, the amplitude of the late negativity (Nc) in response to novel auditory and visual stimuli appears to correspond to the synaptic density in the prefrontal cortex, which is maximal at about 2 years of age and gradually decreasing thereafter (Courchesne, 1990). Similarly, auditory cortex reaches its maximal synaptic density at about 3 months of age (Huttenlocher and Dabholkar, 1997), the same period when the PC to sounds reaches its highest amplitude (Kushnerenko et al., 2002a,b). As illustrated by the above examples (as well as those discussed in Section Studying clinical and at risk groups), currently, this approach cannot not go beyond generalities in explaining the development of ERP measures. For more specific explanations, more detailed information would be needed about the effects of maturational changes in the brain on the large-scale electric activity measurable from the scalp.

#### Interactive specialization

This is a constructivist viewpoint that assumes that the postnatal functional brain development involves activity-dependent processes that will organize patterns of interregional interactions, leading to cortical specialization. For instance, biases in passive auditory attention during the first year of life, e.g., being highly sensitive (as opposed to being moderately sensitive) to distraction by deviant sounds, will be reinforced by different experiences and will lead to differential patterns of cortical specialization in childhood and adulthood. For example, as we discussed in Section Studying clinical and at risk groups, persistent distractibility to sounds that are not behaviorally relevant may hinder the development of typical cortical specialization in other areas (e.g., learning to speak). This approach can help in clarifying the concepts and theories regarding critical periods and brain plasticity, open new lines of research linking the processes of passive attention (and ERP responses as possible measures) to environmental and interpersonal variables of interest to developmental psychologists, as well as provide the basis for setting up intervention strategies and methods for testing the effectiveness of the intervention (Johnson, 2005).

#### Skill learning

This view proposes a continuity of the brain mechanisms of cognition throughout the whole life span. Thus, for example, the brain regions that are active in infants during the onset of auditory attention are assumed to be similar or even identical to those involved in the same, though more complex skills in adults. While it remains unclear whether parallels can be drawn between adult experience and skill learning in infants, the findings regarding both general auditory (e.g., Smith et al., 2006; He and Trainor, 2009; Gervain and Werker, 2012) as well as musical (e.g., Phillips-Silver and Trainor, 2005; for a review, see Trainor et al., 2012) and language skills (e.g., Cheour et al., 2002b; Dehaene-Lambertz et al., 2002, 2010; Teinonen et al., 2009; for a review, see Dehaene-Lambertz et al., 2006) point in this direction. Although this appears to be a fruitful approach, we know of no studies explicitly testing skill-learning related hypotheses for the processing of sounds with large acoustic deviations in young infants. More evidence has been obtained from older children. For example, distraction by irrelevant sounds may be stronger in 10 year olds than that seen in adults (for a review see, Werner, 2007) and preventing distraction by available predictive cues does not reach full maturity until adulthood (Wetzel et al., 2009). However, the strong version of this hypothesis regarding the relative stability of the neural substrate remains to be tested. In sum, this approach may provide a framework for future research into the neural processes underlying passive attention and orienting in young infants.

Finally, although to our knowledge, no studies have yet been published relating the ERP components of passive auditory attention in young infants to genetic factors, this line of research has much to offer to the field. In adults, Heitland et al. (2013) found evidence for the involvement of frontal-cortical dopaminergic and serotoninergic mechanisms in eliciting the P3a response. Other recent studies then described effects of polymorphisms in dopamine and serotonine genes on distractibility, inhibition, and visual disengagement in young infants (Holmboe et al., 2010; Johnson and Pasco Fearon, 2011; Leppänen et al., 2011). For instance, Holmboe et al. (2010) found correlations between variants of some dopamine-system genes and distractibility in a visual attention task in 9 month old infants. In 7 month old infants, Leppänen et al. (2011) found correlation between performance in disengaging attention from a central (in the fovea) stimulus to a target presented at the periphery and polymorphisms of a serotonin-system gene. This visual “disengagement' capacity” appears to be a sensitive marker of typical development and infants with difficulties in shifting their gaze away from the stimulus currently at the fovea are at risk for ASD (Zwaigenbaum et al., 2005). One might draw parallels between visual disengagement and separating novelty from wide acoustic deviance. Further, in 6-year old children, Birkas et al. (2006) found effects related to polymorphisms of a dopamine-system gene on the LN (N2c) and on resistance to behavioral distraction. Thus, it would be interesting to test how dopamine- and serotonin-system related polymorphisms are related to the various processes of auditory passive attention in infants. As Johnson and Pasco Fearon (2011) argued, gathering data on genetic variation provides new possibilities for separating components of the infant mind in general and the processes of passive attention in particular. Combining ERP measures for irrelevant sounds with genetic information may provide insights into the mechanisms and functional properties of the early emerging abilities of auditory information processing. Specifically, although the human brain is already organized at birth (Dehaene-Lambertz, 2011), maturation occurs over a protracted period and, in addition to endogenous changes, stimulus-driven neural activity also influence the development of connectivity in the brain (Del Rio and Feller, 2010; Tau and Peterson, 2010). We can thus conclude that, although “a bias for specific input characteristics” is in place at birth—as our review also showed—it is the interplay between genetic and environmental factors that will drive development of attention over time (Scerif, 2010).

## Summary

We reviewed the—as of yet—rather patchy evidence available on the development of processing wide acoustical deviance and contextual novelty in young (<1 year old) infants. We found some evidence suggesting that even newborn infants process these two types of information somewhat differently, but also that separation between them emerges gradually and proceeds beyond the first year of life. Further, the ERP components associated with deviance detection, attention-switching/distraction, and reorientation in adults may have their roots in the waveforms elicited in young infants. Due to the relatively high reliability of the ERP responses to acoustically widely deviant sounds in young infants, this paradigm may serve well the purposes of research into individual variability, clinical and at-risk groups. However, although a handful of studies have already been published on these topics, most of this research lies in the future, just as the establishing of the neural and gene tic bases of the development of passive attention.

### Conflict of interest statement

The authors declare that the research was conducted in the absence of any commercial or financial relationships that could be construed as a potential conflict of interest.

## Footnotes

^1^Although it has long been known that sounds are processed even before birth (e.g., Ray, 1932; Hepper and Shahidullah, 1994) for the full timeline of development, see (Moore and Linthicum, 2007), no systematic study testing the processing of wide acoustic deviance has been published yet.
